# Ruptured Aortic Aneurysm and Dissection Related Death: an Autopsy Database Analysis

**DOI:** 10.1007/s12253-020-00835-x

**Published:** 2020-06-16

**Authors:** Dániel Pál, Brigitta Szilágyi, Márton Berczeli, Csaba Imre Szalay, Balázs Sárdy, Zoltán Oláh, Tamás Székely, Gergely Rácz, Péter Banga, Zsófia Czinege, Péter Sótonyi

**Affiliations:** 1 Heart and Vascular Center, Department of Vascular and Endovascular Surgery, Semmelweis University, Városmajor u. 68, Budapest, 1122 Hungary; 2 Department of Geometry, Institute of Mathematics, Budapest University of Technology and Economics, Budapest, Hungary; 3 Heart and Vascular Center, Department of Cardiology, Semmelweis University, Budapest, Hungary; 4 2nd Department of Pathology, Semmelweis University, Budapest, Hungary; 5 1st Department of Pathology and Experimental Cancer Research, Semmelweis University, Budapest, Hungary

**Keywords:** Acute aortic syndrome, Aortic dissection, Aortic aneurysm, Autopsy, Rupture, Bleeding

## Abstract

Acute aortic catastrophes (AAC), mainly ruptured aneurysms and dissections, lead all other vascular conditions in morbidity and mortality, even if intervention occurs. The aim of our study was to give a descriptive overview of the demographic and pathological characteristics of AAC. Between 1994 and 2013, 80,469 autopsies were performed at Semmelweis University hospitals in Budapest. After collecting the autopsy reports we were able to create the AAC database upon which we conducted our analysis. We found 567 cases of AAC. The cause of death in 120 of them was classified as a non-ruptured aorta with malperfusion or distal embolization. Of the remaining 447 cases, in 305 the cause of death was a ruptured aortic aneurysm (rAA), and in 142 it was a ruptured aortic dissection (rAD). The distribution of rAA cases was 34.4% thoracal, 4.3% thoracoabdominal, and 61.3% abdominal. We found female dominance where the rAA was thoracal. In rAD cases, 84% were Stanford A and 16% Stanford B type. In both groups we found different pathological distributions. In the prehospital group, the number of thoracal ruptures was considerable. 88% of the patients with Stanford A dissection died in the prehospital or perioperative period. The most progressive AACs were ruptures of intrapericardial aneurysms and Stanford A dissections., however survival rate can be elevated by using rapid imaging examination and immediate surgical intervention. We want to highlight that our study contains such gender differences, which are worth to be taken into consideration.

## Introduction

Cardiovascular diseases are the leading cause of death in Hungary [1]. Among them, AACs caused by aortic rupture are relatively rare, but they are severe conditions with high mortality. Most frequently, AACs are caused by rAA and rAD.

Aortic aneurysms form in both the abdominal and thoracal segments. There are gender differences recorded in the literature [2, 3], which we consider in our analysis. Aortic aneurysm formation occurs most frequently in the abdominal segment – almost 9 times more frequently than in the thoracic segment [4]. According to multiple studies, the prevalence of abdominal aortic aneurysm (AAA) ranges from 1.3–12.7% in the elderly population, with a male to female ratio of (2–4):1 [5–7]. The incidence of ruptured AAAs is 1–21 cases per 100,000 persons/year, with a male-female ratio of (6–9):1 [6, 8]. They are not rare, however, and show an increasing incidence, 59 to 104 cases per 1,000,000 persons/year [9, 10]. Nowadays, screening for AAAs is recommended for high-risk groups such as male smokers over 65 [11]. The distribution of thoracal aortic aneurysms TAAs is as follows: 40% on the ascending segment, 15% on the aortic arch, 35% on the descending segment, and 10% are thoracoabdominal aortic aneurysms ThAAs [10]. Early diagnosis of aortic aneurysms is crucial for successful treatment.

Acute aortic dissection (AAD) is one of the most common catastrophic events affecting the aorta, with an incidence of (29–35)/1,000,000 persons/year [12, 13]. The intimal tear is located in the ascending aorta in 65% of the cases, the descending aorta in 25%, and in the arch or the abdominal aorta in 10% [14]. The incidence of Stanford A and B type dissections peaks at 50 and 60 years, respectively. The male-to-female ratio has been reported to be 4:1 [14, 15]. Depending on the affected segment, the most common complications are aortic rupture, cardiac tamponade, aortic regurgitation, thromboembolism, and the compression of coronary or peripheral arteries [16, 17]. The diagnosis of AAD is difficult because the possible symptoms can mislead the physician to other kinds of pathologies, such as myocardial infarction or stroke. The mortality rate of hospitalized but untreated Stanford A dissection patients can be as high as 50–58% [14, 18].

The aim of our research was to investigate the occurrence of aortic ruptures leading to death using the medical autopsy records of the institutes of Semmelweis University, to describe the most specific clinicopathological parameters of AAC, and to investigate certain demographic and morphological features regarding cases with aortic aneurysm rupture or acute dissection leading to rupture.

## Materials and Methods

We collected autopsy data from the records of Semmelweis University’s (Budapest, Hungary) 1st Department of Pathology and Experimental Cancer Research, 2nd Department of Pathology, and the Department of Forensic and Insurance Medicine for the 20-year period from 1994.01.01 to 2013.12.31. These institutes perform autopsies in cases when sudden death occurs in public institutions and public spaces in the entire area of Budapest and Pest County, as well as on the premises of Semmelweis University. Budapest and Pest County are home to about 25% of the Hungarian population [19]. During the 20-year period, 80,469 autopsies were reported. We analyzed every case where the “cause of death” or the “diagnosis related to the patient’s death” was defined as aortic aneurysm or aortic dissection. We collected these cases using the International Classification of Diseases, Tenth Revision, Clinical Modification (ICD-10-CM) code (aortic dissection: I7100-I7103 and aortic aneurysm: I711-I718).

Based on the resulting list, we checked the medical records for each case and created a database with the following data:

- ** Demographics**: age, gender, hospital-admission information, date and place of death,
- **Type of aortic pathology**: aortic aneurysm (AA) or aortic dissection (AD),
- **Localization of the lesion**: ascending, arch, descending, thoracoabdominal, suprarenal, infrarenal, or combined,
- **Extent of the aortic dissection**: Stanford A or B type, and
- ** Direction of the bleeding (if rupture occurred)**: thorax, pericardium, bronchi, esophagus, abdominal cavity, cava, or retroperitoneum.

Then we organized the population into groups and analyzed the data. We divided the total population into three groups according to hospital admission and therapeutic intervention: Group I suffered pre-hospital death, group II suffered pre-intervention hospital death, and group III suffered peri- or post-intervention death. We also divided the population into two groups according to the two major complications of AAC that lead to death: ruptured acute aortic catastrophe (rAAC) or non-ruptured acute aortic catastrophe (nrAAC). Finally, we analyzed the yearly and monthly distribution of cases, the demographic data, the clinical course of each case, the place of death (home, public space, ambulance, or hospital), and the place of medical care (emergency department, intensive care unit, general surgery department, or cardiovascular surgery department).

## Results

**Fig. 1:**
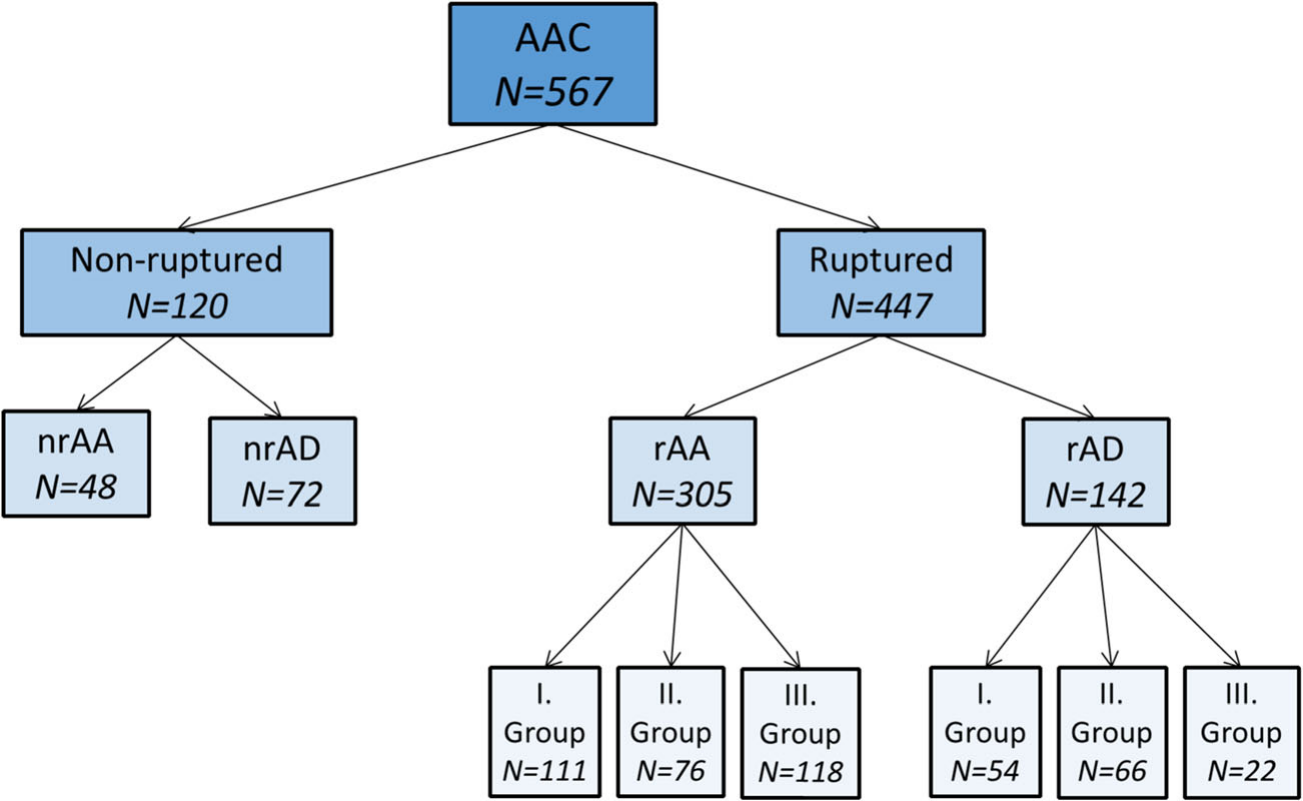
Acute aortic catastrophes, 1994-2014, N=567

Of the 80,469 recorded autopsies, aortic aneurysm or dissection was the cause of death in 567 (0.7%) cases. Of these 567 AAC cases, 447 (79%) belong in the rAAC group, while 120 (21%) in the nrAAC group. In the nrAAC group, the aortic event leads indirectly to the patient’s death (malperfusion by intimal flap or distal embolization – stroke, mesenteric ischemia, myocardial infarction, respiratory insufficiency, acute renal failure, etc.). The rAAC group, where the aortic bleeding leads directly to the patient’s death, is further divided into two subgroups: ruptured aortic aneurysm (rAA) in 305 cases (68.2%) and ruptured aortic dissection (rAD) in 142 cases (31.8%) (Fig. 1).

### Non-ruptured AAC Group

Two thirds of the lethal complications occurred in the nrAD group. The most common causes of death were acute heart failure, acute mesenteric ischemia, and preoperative stroke. In 56 cases (47% of the nrAAC group), we found distal embolization. We defined malperfusion syndrome as a complication caused by branch-vessel involvement and resulting in end-organ ischemic dysfunction by folating intimal flap in 64 cases (53% of the nrAAC group). This was verified by pathological examination (macroscopic and histological) **(**Table 1).

**Table 1:** Analysis of non-ruptured AAC cases (N=120)

Distal occlusions (N=120)	N	Total (%)
Coronary	9	8
Supraaortic branches	6	5
Visceral branches	24	20
Lower limb artery	8	7
Combination	9	8
Occlusion by intimal flap	64	53
Cause of death (N=120)	**N**	Total (%)
Acute heart failure, acute myocardial infarction	61	51
Hemorrhagic shock, consumption coagulopathy	3	3
Acute ischaemic colitis, peritonitis	13	11
Acute renal failure	1	1
Pulmonary embolism, ARDS	5	4
Stroke	9	8
Intraoperative, irreversible, acute circulatory failure	11	9
Postoperative sepsis	12	10
Postoperative, irreversible circulatory failure, ARDS	5	4

### Ruptured AAC Group

Patients who died as a result of rupture and congestive bleeding form a homogenous and large-scale population. We focused on this group and eanalyzed 447 ruptured cases. In the examined institutes of Semmelweis University, the yearly average of the autopsy cases were: 15 for rAA and 7 for rAD. During our twenty years-long followup, the number of cases showed an increasing tendency, while the overall number of autopsies remained unchanged. There was an increase in the rAA group, from 3.27 to 4.29 of 1,000 autopsies. In the rAD group autopsies also increased, from 1.42 to 2.02 of 1,000.

### Demographic Data

**Fig. 2:**
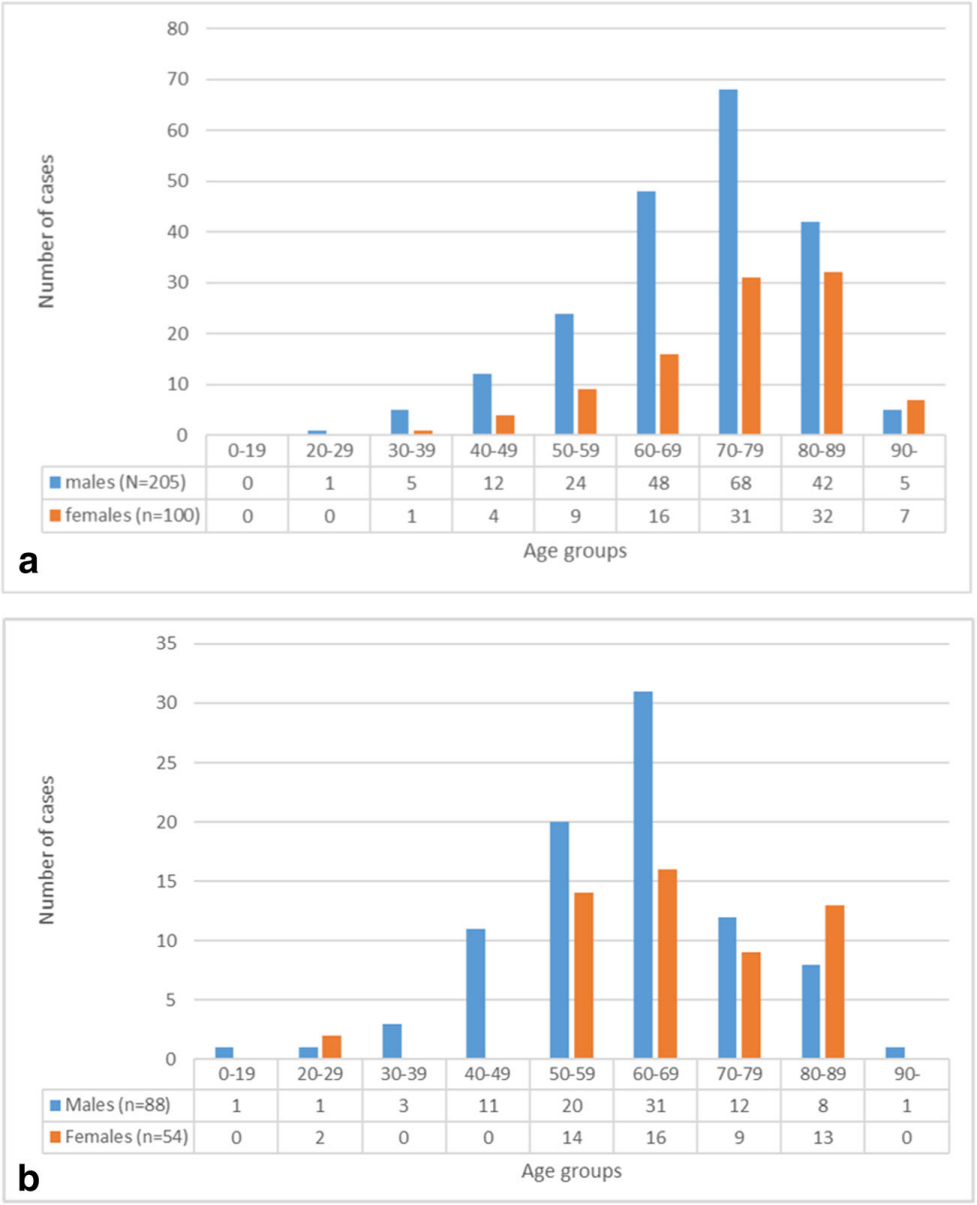
**a** Age and gender differencies in rAA group, 1994-2014, N=305. **b** Age and gender differencies in rAD group, 1994-2014, N=142

The average age at the time of death was 71.0 ± 13.1 years in the rAA group. In the rAD group, it was 62.6 ± 14.5 years. The male/female distribution in the rAA group was 21:10 and 3:2 in the rAD group. The proportion of female pacients increased in older age groups. At the second decade of our examined period, average age increased from 70.0 ± 11.8 (N = 126) to 71.7 ± 13.9 (N = 179) in the rAA group, while in the rAD group, it decreased from 63.8 ± 14.8 (N = 56) to 62.4 ± 14.4 (N = 83). The youngest patient with rAA was 28 years old, while at the case of rAD the youngest patient was 14, futhermore the oldest patient with rAA was 100 years old and with rAD the oldest one was 94 (Fig. 2a and b).

### Clinicopathology

**Fig. 3:**
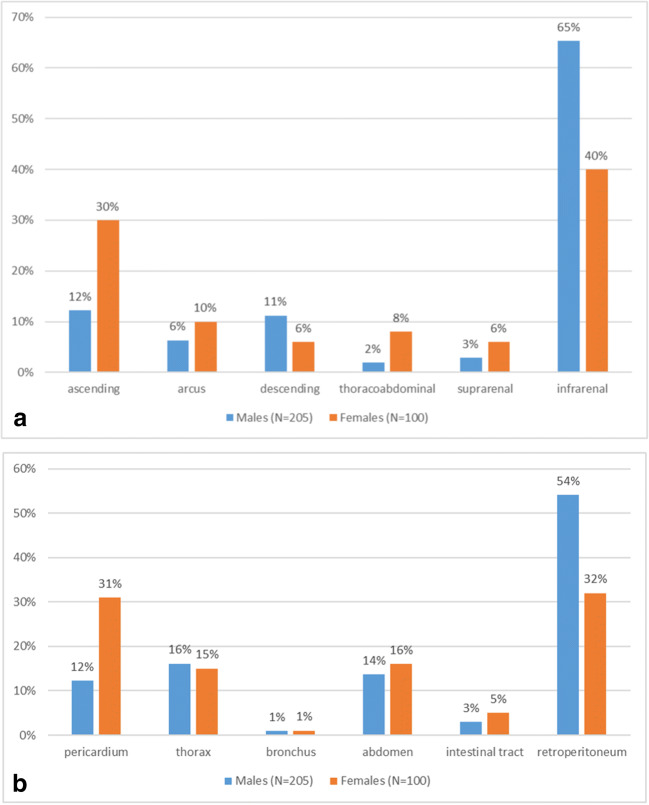
**a** Gender differencies in rAA localisation, 1994-2014, N=305. **b** Gender differences in direct of rupture, rAA group, 1994-2014, N=305

Regarding the localization of the aortic aneurysms, the autopsy reports showed that 61.3% of the aneurysms were abdominal, 34.4% were thoracic and 4.3% were thoracoabdominal. As we disaggregated the cases by gender, we found a difference. In men, there was a balanced proportion of all three regions, while for women the ascending region dominated. Chest location was significantly higher among women (total chest lesions in females were 46% and males 28.8%) (Fig. 3a and b)

In the rAD group, the rate of Stanford A type cases was much higher than Stanford B types. Thoracic aneurysms and the Stanford A dissections predominantly bled in the direction of the pericardium or the thoracic cavity. We did not find localization or gender difference in the rAD group (Table 2).

**Table 2:** rAD clinicopathology, 1994-2014 (N=142)

Types	Direction of rupture					
rAD cases (N=142)						
	Bronchus	Thorax	Pericardium	Abdomen	Retroperitoneum	Sum (%)
A	0	12	107	0	0	119 (84)
B	1	11	4	2	5	23 (16)
Sum (%)	1 (1)	23 (16)	111 (78)	2 (1)	5 (4)	
Males (N=88)						
A	0	8	66	0	0	74 (84)
B	0	5	3	1	5	14 (16)
Sum (%)	0 (0)	13 (15)	69 (78)	1 (1)	5 (6)	
Females (N=54)						
A	0	4	41	0	0	45 (83)
B	1	6	1	1	0	9 (17)
Sum (%)	1 (2)	10 (19)	42 (78)	1 (2)	0 (0)	

### Clinical Course

Analyzing the place of death revealed that patients in both groups had a similar chance of admission into a health care institute 63.6% for rAA (N = 194) and 62.0% for rAD (N = 88). For the rest of the patients, death was diagnosed in public spaces, at home (rAA 28.9%, rAD 32.4%), or during transportation to a health care institute (rAA 7.5%, rAD 5.6%).

Among those cases in which the death occured in a health care institute, 70.6% of rAA cases reached the cardiovascular surgery department, 15.5% reached the emergency department, 9.8% were in other surgical departments, and 4.1% were in the intensive care unit. 52.3% of the hospitalized rAD cases reached the cardiovascular surgery department, 34.1% reached the emergency department, 5.7% were in other surgical departments, and 8.0% were in the intensive care unit.

Based on the place of death we created three groups. then we conducted a more detailed pathological analysis of these groups. In the prehospital group (Group I), the largest number of the cases was retroperitoneal, but the case number of ascending aorta ruptures were also considerable (13% out of 305 cases). In the perioperative (Group II) and postoperative (Group III) groups, the direction of bleeding was retroperitoneal in almost one third of the cases (32%) (Table 3).

**Table 3:** Pathological differences depending on the place of death, rAA group, 1994-2014 (N=305)

The location of the rAA							
	I. group		II. group		III. group		Sum
	N (%)	in subtype %	N (%)	in subtype %	N (%)	in subtype %	N (%)
Ascending	39 (13)	71	9 (3)	16	7 (2)	13	55 (18)
Arcus	9 (3)	41	7 (2)	32	6 (2)	27	22 (7)
Descending	8 (3)	29	7 (2)	25	13 (4)	46	28 (9)
Thoracoabdominal	1 (0)	8	5 (2)	38	7 (2)	54	13 (4)
Suprarenal	4 (1)	33	3 (1)	25	5 (2)	42	12 (4)
Infrarenal	50 (15)	29	45 (14)	27	80 (23)	44	175 (57)
Sum	111 (36)		76 (25)		118 (39)		
The direction of the rupture							
Pericardium	41 (13)	73	10(3)	18	5 (2)	9	56 (18)
Thorax	13 (4)	27	11 (4)	22	25 (8)	51	49 (16)
Bronchus	2 (1)	67	1 (0)	33	0 (0)	0	3 (1)
Retroperitoneum	42 (14)	30	36 (12)	26	63 (21)	45	141 (46)
Abdomen	13 (4)	29	14 (5)	31	18 (6)	40	45 (15)
Intestinal	0 (0)	0	4 (1)	36	7 (2)	64	11 (4)
Sum	111 (36)		76 (25)		118 (39)		

I. group: prehospital cases (N=111)

II. group: preoperative cases (N=76)

III. group: perioperative and postoperative cases (N=118)

74% of the Stanford A type rADs were found in the prehospital and perioperative groups. The most frequent direction of rupture bleeding was pericardial, and over one third of these patients died before receiving medical attention, while another third of them died before adequate surgical intervention could have been performed (Table 4).

**Table 4:** Pathological differences depending on the place of death, rAD group, 1994-2014, (N=142)

The location of the rAD							
	I. group		II. group		III. group		
	N (%)	in subtype %	N (%)	in subtype %	N (%)	in subtype %	Sum (%)
Stanford A	48 (34)	40	57 (40)	48	14 (10)	12	119 (84)
Stanford B	6 (4)	26	9 (6)	39	8 (6)	35	23 (16)
Sum (%)	54 (38)		66 (46)		22 (15)		
The direction of the rupture							
Pericardium	50 (35)	45	50 (35)	45	11 (8)	10	111 (78)
Thorax	2 (1)	9	13 (9)	57	8 (6)	35	23 (16)
Bronchus	0 (0)	0	1 (1)	100	0 (0)	0	1 (1)
Retroperitoneum	1 (1)	20	1 (1)	20	3 (2)	60	5 (4)
Abdomen	1 (1)	50	1 (1)	50	0 (0)	0	2 (1)
Sum (%)	54 (38)		66 (46)		22 (15)		

I. group: prehospital cases (N=54)

II. group: preoperative cases (N=66)

III. group: perioperative and postoperative cases (N=22)

## Discussion

A large number of studies focus on AACs, but most of these concentrate on the diagnosis and treatment. Meanwhile, our aim was to describe the most important clinicopathological features of these high mortality disorders. In formerly published literature, we found a similar publication [20]; however, a clear comparison cannot be made, due to changes in understanding, classification, historical and demographic aspects, as well as changes in the regulations concerning autopsies.

Worldwide, approximately 0.5% of all deaths is caused by aortic aneurysm and dissection, and according to some research, the proportion may increase [21]. Our findings reveal a similar proportion in our autopsy database [22]. We want to highlight, whoever died suddenly in Hungary, his or her autopsy was done in any case. Therefore, we got a full and accurate overview of the mortality data in this Hungarian region. Our data shows that over two thirds of ruptured cases resulted by aneurysm, while non-ruptured cases are linked predominantly to dissections. Of the non-ruptured subgroup of the AAC population, side branch occlusion occurs in about 47% of the cases (N = 57), which is around 10% of our population (N = 567). In published literature, we found this to be 15–40% [23, 24]. The low values can be explained by our choice to analyze autopsy cases only. Proving the presence of ischemia and consequential malperfusion is a difficult task even if it based on both autopsy and clinical data. The results are uncertain without the use of specialized imaging methods (MRA, CTA) or histological examination. For this reason, critical judgment and interpretation are required.

During the examined 20 year-long period (1994–2014), both the average age and the incidence increased in these two groups. Due to age distribution, at the age of 80–84, the number of the cases increased by 48% and at the age above 85 years, the growth is 92%. This would explain why we noticed a relation between the rising number of cases and age distribution. Based on these results, we believe that the examined population is an aging population. Wang et al. reported a similar tendency. They examined the autopsy reports of patients (N = 909) who died as a result of aortic aneurysm, at the ages between 60 and 100 [22]. The results showed that AA is significantly more frequent among people at the age of 80 and above. The average age decreased only in the rAD group. This may be explained by the Central European paradox that describes an incidence increase of ever-earlier fatal cardiovascular complications (e.g. AMI) of hypertension, chronic stress and smoking [25]. The increase in the proportion of women in older age groups can be explained by the fact that women usually live 6–8 years longer [1].

Former studies reported that 60–70% of rAA cases affected the abdominal part, while 2030% affected the thoracic area, and 3–5% the thoracoabdominal area. These findings are similar to our results [26, 27]. Earlier studies also drew attention to gender differences, although men have more aortic aneurysms but women with chest lesions are more common and the risk of rupture is greater, therefore they have a higher prehospital mortality rate. This has been explained by several molecular pathological and pathophysiological (role of oestradiol-receptor expression and circulating oestradiol-level, elastin/collagen ratio) researches, animal experiments, but further human studies are needed [28–30]. According to our findings, in the prehospital group, the mortality of rAA showed dominance at the case of thoracic ruptures, due to the fulminant progression of the disease, which causing rapid pericardial bleeding and cardiac tamponade. At the same time, the tamponading effect of the crura of the diaphragm and the retroperitoneum can cause the deceleration of the progression the abdominal ruptures.

Previous clinical observations show a two thirds to one third ratio of Stanford A and B types of aortic dissection [14], while our study shows a much higher Stanford A:B ratio (84:16). The dominance of type A dissections can be explained with the studied population, because the fulminant progression of Stanford A dissections are overrepresented in the autopsy population.

According to our study, 62% of rAD and 63.6% of rAA cases reached the hospital. Prehospital death rates for these groups were 38% and 36.4%, respectively. Clinical studies of rAA showed differing results, depending on the type of aneurysm. According to Reimerink et al., 27% of abdominal rAAA patients reached a hospital [31]. Johansson et al. reported that 47% of ruptured thoracic aortic aneurysms patients reached a hospital [32]. Our study found an approximation of 60% hospitalization rate. This result applies to the total aneurysms of the full length aorta, not just one segment, so we did an autopsy database analysis. It was difficult to compare it with the clinical data. Melvinsdottir IH et al. reported in their retrospective study that acute aortic dissection patients had a 17.6% prehospital mortality [33]. Axelsson C. et al. reported that in 2010, in Göteborg, 78% of acute rAD patients reached an emergency department [34]. Mészáros et al. conducted a population-based longitudinal study in Hungary between 1972 and 1998 regarding the epidemiology and clinicopathology of aortic dissections. According to their study, preoperative mortality was between 21% and 79% for patients who reached a hospital [13].

We want to highlight that, in our study population, 74% of the rAA and 44% of the rAD cases reached the department of vascular or cardiac surgery. Diagnostic delays and faster rAD progression were difficult to separate in this research. We did not find any clinical data, it was only an autopsy database analysis.

There is a correlation with the prehospital (1), preoperative (2) and peri/postoperative (3) groups and the onset of the symptoms and the progression of the disease. In regards to the rAA cases, we reported that in the prehospital (N = 110) and in the perioperative (N = 75) groups, the number of ascending aorta aneurysms was significantly higher than the number of infrarenal aortic aneurysms. Bleeding into the pericardiac space after rupture was also significantly more frequent in the prehospital and preoperative groups than bleeding into the retroperitoneal space. Stanford A cases were also significantly more common in the prehospital and the preoperative groups than in the peri/postoperative group. These findings also correlate with the high percentage of pericardiac tamponade in the first two groups. It is a well-known fact that pericardiac tamponade rapidly leads to death. In the peri/postoperative group, there were no other significant differences in the number of Stanford A and B cases.

Our explanation for the differing results is that we analyzed an autopsy-based population. In Hungary, Emergency Departments began to function only in the past 10–15 years, while the varied clinical symptoms of aortic dissections make it harder to arrive at the correct diagnosis. There was a similar result in misdiagnosis rates in our aortic dissection population. We believe that the time factor was the answer to the question: why people with rAD could not reach a cardiovascular department.

## Conclusions

In conclusion, we can declare that analyzing pathological data provides useful information for clinicians. It points out diagnostic mistakes, the difficulty of patient care and helps with prognostic judgment. The acute aortic dissections and thoracic aortic aneurysm ruptures are likely to lead fatal pericardial or chest bleeding. Therefore, these patients have lower chance to adequate care. Our results also show that the survival rate of chest aorta aneurysm catastrophes can be increased by rapid imaging examination (CT, MR) and an immediate surgical intervention. In cases of the abdominal aortic aneurysms a kept rupture is more frequent, which causes less cardiovascular instability, therefore more time is available to establish the diagnosis and treatment. Elective interventions have the best outcomes, so screening and planned surgeries would be considered the best treatment. From the formerly mentioned statement, a question crops up: Is the regular screening for aortic aneurysm recommended in special selected populations – male, smoker, above 65 years? There is no obvious answer, either pro or con. Our results give further impetus to the sex differences in human research, as well as contribute to the development of detailed screening programs. Our research confirms that in an aging society with rising life expectancy the number of the cases is going to increase. That is the reason why we should pay attention to and promote the screening programs for patients who are exposed to risk factors or have a family history of acute aortic catastrophes. The prevention and screening is a great chance (risk factor reduction and early diagnosis) for these patients.

## Study Limitation

The three institutes use different database, and they have changed several times over the 20 years. Those factors made it difficult to create a complete and consistent database. In the comparison of an autopsy-based study and a clinical-based study we have to be exact, because the examined populations are not the same just overlapping. Otherwise the data of the topic differ from each other which makes the comparison cumbersome. Our 20-year study’s limitation is the examined population, we can not use correctly the concept mortality and incidence, because all our data is based upon autopsy reports.

## Abbreviations

AAC: Acute aortic catastrophes
AAA: Abdominal aortic aneurysm
rAA: Ruptured aortic aneurysm
rAD: Ruptured aortic dissection
TAA: Thoracal aortic aneurysm
ThAA: Thoracoabdominal aortic aneurysm
rAAC: Ruptured acute aortic catastrophe
nrAAC: Non-ruptured acute aortic catastrophe
